# Sexual behaviour and sexual and reproductive health education: a cross-sectional study in Romania

**DOI:** 10.1186/1742-4755-11-48

**Published:** 2014-06-23

**Authors:** Cornelia Rada

**Affiliations:** 1 Biomedical Department, “Francisc I. Rainer” Anthropology Institute of the Romanian Academy, 8 Avenue Eroii Sanitari, O.P. 35, C.P. 13, Sector 5, 050474 Bucharest, Romania

**Keywords:** Sexual behaviour, First intercourse, Virginity, Knowing one’s first sexual partner, Safe sex, Condom use, Sex education, Sexual and reproductive health, Socio-demographic characteristics, Romania

## Abstract

**Background:**

Up-to-date, genuine sexual and reproductive health (SRH) education programmes have been possible in Romania only since communism collapsed in 1990. Since 2006, Romania has had no national strategy in this field. Under current global circumstances (high labour mobility, internationally mixed marriages), issues previously considered solely national have become worldwide concerns.

**Methods:**

In 2011–2012, 1215 respondents homogeneously distributed on background, gender, educational level and age group (18–74) were sampled. This article uses a 96-item questionnaire about family and SRH, presenting results on nine items: first intercourse (FI), virginity, knowing first sexual partner, safe sex, number of sexual partners and sexual education. The data were analysed using Pearson chi-square tests and latent class analysis.

**Results:**

Some participants (7.2%) engaged in FI at age 15 or earlier. The average age at FI was lower for men (18.08), for individuals with a lower education level (18.07) and for those in rural areas (18.27), compared with that for women, those with more education and those in urban areas, respectively. The average age at FI was over 2.5 years lower for people aged 18–24 (16.99) than for those aged 60–74 (p < 0.001). More than 60% were not married or partnered with their FI partner, and 17.8% engaged in FI less than a month after meeting their partner. Less than one-fourth practiced safe sex at FI, with higher proportions for the urban sample, those with an average level of education and those aged 18–35 (p < 0.001). Higher average numbers of sexual partners were found among men (6.56, compared with 2.37 among women), in urban areas (5.07, compared with 3.75 in rural areas) and among those with higher levels of education (p < 0.005). On average, subjects first received information on SRH at 15.39 years of age, with only 10% listing the school, doctors or medics as a source.

**Conclusions:**

Unsafe sex, early initiation of sexual activity and poor SRH education from schools, experts and parents require a multidisciplinary approach within prevention programmes, especially among the populations at risk: rural residents, those with low levels of education and youth.

## Background

Although there is no precise age in this respect, it is estimated that the optimum age for becoming sexually active, both in boys and in girls, is 18–22 years old. This range reflects the age of physical and mental maturity, although the maturation of the sexual organs begins earlier (at 9–14 for girls and 12–18 for boys). The risks of early initiation of sexual activity (before the end of puberty, for girls until around age 16 and for boys until around age 16 or 17) can be cervical cancer, breast cancer, early aging, infertility and infection with human immunodeficiency virus (HIV) and other sexually transmitted infections (STIs). Youths who become sexually active too early are in danger of having more sexual partners, using condoms to a lesser extent and engaging in other health risk behaviours including alcohol consumption, smoking, drug consumption, delinquency and violence [1,2]. Sexual initiation after the optimum age range presents risks such as ovarian and testicular cysts and the development of withdrawn, shy or neurotic behaviour [3-5]. Teenage girls who start their sexual activity too early and who have higher numbers of non-marital sexual partners run a higher risk of contracting STIs, having unplanned pregnancies, becoming single mothers, having more abortions and experiencing marital instability and poverty. In the long run, early sexual initiation increases the chances of engaging in risky sexual activity. This risky behavioural script also affects sexual functioning as well as the capability for a relationship in adulthood [6].

The timing of sexual initiation is influenced by biological aspects such as menarche as well as pollution, but especially by culture, environment, society and generally the world in which the individual lives. Thus, regarded as a life transition, sexual initiation decisions are influenced by the models of peers and parents. Sources of information on the extensive subject of reproductive sexual health have a particularly important role in the formation of the concepts of sex, sexuality and gender and in the development of sexual behaviour.

A study conducted in 44 countries in 2005 and 2007 found that the average age at first intercourse (FI) was 18.4 years. In Norway, Sweden and Finland, the average age at FI ranged from 16.2 to 16.5 years, while and in India, Nepal and China, it was between 22.1 and 22.9 years [7].

The first sexual experience is a rite of passage to maturity and is experienced as an important event about which most people have memories. Particularly significant is the loss of virginity, the importance and manner of realization of which differs by gender [8]. An in-depth interview study conducted in 2002 with 61 American respondents aged 18–35 identified three categories of description of first sexual experience. Approximately half of the subjects regarded their virginity as something valuable to offer to someone dear and were willing to wait until they found the right person worthy of this gift. More than a quarter regarded their virginity as a stigma they had to get rid of as soon as possible, the loss of virginity being necessary to maintain social status and to assert a gender identity. This group had an earlier sexual initiation and tended to share their first sexual experience with less knowledgeable people. The rest regarded the loss of virginity as an important and inevitable rite of passage to maturity, for which it was important to achieve a good management of the physical, social and emotional risks associated with the loss of virginity. These findings regarding the perception of the first sexual act are important for public health policy [9-11].

Waiting until you find the right person with whom to have sexual relations, in general, and for FI in particular, decreases the risk of being hurt by unsuitable partners. Being regarded as a part of the growth process, having FI with a reliable partner increases the chance of having safe sex. Earlier initiation of sexual activity can lead at first to negative feelings of embarrassment because of the lack of experience and can involve a high risk of STIs as a consequence of the lack of protection [12].

Consistent sex (1–3 times a week) in a harmonious relationship of love and attachment, not with occasional partners, is associated with health benefits including increasing the lifespan, easing various pains, burning calories, maintaining physical attractiveness, regulating menstruation, increasing calmness, facilitating tolerance and decreasing anxiety [13-15].

### Sexual behaviour and sexual and reproductive health (SRH) in Romania

As was the case for many formerly communist countries, Romania was closed to influences from the West under communist rule. Communist mass media monitored all publications, and books about sexuality were limited to covering politeness between spouses and STIs. Before December 1989, when the communist regime was replaced in Romania, it was shameful for an unmarried woman to get pregnant, for unmarried men and women to live together as a couple and for a girl to have sex before marriage. Although society had grown more permissive, especially with respect to women being virgins when they got married, even as late as the 1980s, young Romanian women kept secret as much as possible whether they had become sexually active. Sexual education in schools was promoted for the purpose of a so-called ‘sexual emancipation’, but this was limited to topics of anatomy and hygiene. Any movie broadcast in Romania was censored and had all erotic scenes removed [16,17].

Sexual abstinence before marriage was a common traditional custom in Romania, especially in the rural environment. Age models relating to sexual activity, marriage and maternity changed. The change affected both genders, but the shift was stronger for women; as a consequence of their changing status and of emancipation, they became sexually active before marriage and postponed marriage as well as having children. Many causes contributed to this shift, including the weakening of traditional norms, the increase of the educational level, modernization, the increase in population migration from one geographical area of the country to another (and, after 1990, also outside the country) and a more aggressive and more diversified mass media.

In 1990, after the collapse of communism and the establishment of a democratic regime in Romania, the health of women and children was very precarious. These circumstances led to the launch of public health programmes as well as strategies for promoting family planning using modern means of contraception, improving neonatal services, controlling STIs and HIV, screening for genital and breast cancer and advancing sexual education. The results of investigations initiated on reproduction health since 1993 have constituted the basis of national plans and strategies on family planning and sexual and reproductive health.

The Romania Reproductive Health Survey 1999 (RRHS 1999), which included a representative sample of 6888 women aged 15–44 and 2434 men aged 15–49 in Romania, found that 18.3% of the women and 9.9% of the men had not become sexually active. For those between 15 and 19, 74.3% of women and only 54.6% of men had not become sexually active. In the 20–24 age group, 22.3% of women and only 6.7% of men had not become sexually active. In 1999, the median age at becoming sexually active was 19.8 years for women and 17.9 years for men [18].

The ‘Reproductive Health Survey Romania 2004’ (RHSR 2004) includes a chapter dedicated to the sexual behaviour of youth between 15 and 24, based on a sample of 1112 women and 584 men. Over a period of 11 years, the percentage of women who had sexual experience before marriage nearly doubled (from 22.2% in 1993 to 43.6% in 2004). During the same period of time, the percentage of men with sexual experience before marriage remained fairly constant at around 65%. As for the age of sexual initiation, the percentages becoming sexually active earlier than 16 years of age were similar in 1993 and 2004: 7.1 and 7.9%, respectively, for women, and 26.7 and 21%, respectively, for men. It is noteworthy that the study from 2004, in contrast to the 1993 study, showed a delay in the start of sexual life both for boys and for girls [19].

The ‘Demography and Lifestyle of Romanian Women’ (DLRW) study, conducted in 2004 by Metro Media Transylvania with a sample of 1982 Romanian women aged 18–84, found that 6.25% had not yet become sexually active, sexual initiation occurred for 35.2% when they were 19–20 and for 25.2% when they were 17–18. The study also showed that, starting with the cohorts born after 1975, sexual initiation took place increasingly earlier, with the average age being 19 for those born 1975–1979 and 17 for those born 1985–1986 [20].

The Rada–Tarcea study, conducted in 2005–2006 on 1902 urban subjects aged 15–90, found that 9.3% had not become sexually active, with the proportion of women being double than that of men. With 1705 valid responses from subjects of both genders, this study reported an average age at sexual initiation of 18.98 years. The study found statistically significant differences in average age at sexual initiation by gender: 20.05 for women and 17.95 for men [21].

In a comparison of the seven post-communist countries in Central and Eastern Europe (Croatia, the Czech Republic, Hungary, Poland, Romania, Serbia and Slovakia), the teenage pregnancy rate was highest in Romania (34 per 1000 women aged 15–20 [22].

Studies show an under-representation of women from Romania who meet the optimal standards for routine gynaecological examinations and Babes–Papanicolau testing. As a result of the inconsistent presence of national SRH prevention and education programmes, Romania reports an incidence of cervical cancer at least double that of countries such as Finland, Luxembourg and France, where such programmes are consistently available [23,24].

In Romania, the HIV epidemic came to the fore in the early 1990s, owing to HIV being detected in a large number of young children and newborns between approximately 1987 and 1991. In recent years, most new HIV diagnoses have been reported to have been infected through heterosexual contact (65% in 2009). Between 1999 and 2009, the rates of diagnoses of syphilis and gonorrhoea in Romania have been in decline; rates fell from 34.5 to 15.0 per 100,000 people for syphilis (still the highest in Europe) and from 18.5 to 2.9 per 100,000 for gonorrhoea (among the lowest in Europe). Chlamydia remains very poorly reported in Romania [25]. The main problem in terms of STIs is under-reporting. Therefore, it is necessary first of all to establish a prevention strategy and careful monitoring mechanisms in the context of labour migration and the growing sex industry [26].

Since 2006, Romania has had no national strategy on health, sexual rights and reproduction. The National Strategy for Sexual and Reproductive Health in Romania 2012–2015 has not yet been adopted or funded. A coalition of NGOs sent a memo to the Minister of Health of Romania advocating the inclusion of sexual and reproductive health as a priority subfield for the allocation of the European structural funds for 2014–2020, but this step has not yet been taken [27].

### Objectives

Taking into consideration the facts presented above, this study proposes to investigate the age at becoming sexually active, marriage with first sexual partner, time of knowing partner at FI, use of protection at FI, number of sexual partners, age at first receipt of information on sex and sources of information on sexual and reproductive health (SRH). The impact of socio-demographic variables on these items in Romania will also be analysed, and comparisons with other studies will be carried out.

The results of this study fill a gap in the specialised literature on sexual behaviour in Romania. At the same time, the trends identified regarding sexual activity and the sources of information about sex can form the basis of public health programmes targeting SRH.

## Methods

### Design and sampling

This research was conducted using a quantitative cross-sectional study. The sample, which is not representative for the whole country, includes 1215 randomly selected subjects aged 18–74. These age limits were selected taking under consideration the age of majority and the average life expectancy at national level in 2008 (73.03 years overall, 76.68 years for women and 69.49 years for men) [28].

The sample size was computed by taking into account the statistical tests and analyses to be conducted. G*Power software was used for the chi-square test, assuming an effect size of w = 0.4 and 250 degrees of freedom; this produced a recommended sample size of 526 subjects of each gender—a total of 1052 men and women. Owing to the sensitivity of some questionnaire items, the expected rate of missing data in the study was set at 0.10, increasing the sample size required by a factor of 1.10, for a total of 1157 subjects. Accordingly, the study aimed to achieve a target conservative sample size of 1200 subjects. A simple random selection of subjects was undertaken from the sampling frame, which consisted of lists obtained from local governments. I contacted the selected individuals through local government officials, family doctors and university rectors.

The distribution of the subjects was relatively homogenous with respect to environment, gender, educational level and age (Table 1).

**Table 1:** Distribution of subjects according to environment, gender, educational level and age group (socio-demographic characteristics)

**Social-demographic variables**	**Number**	**%**
Environment		
Urban	672	55.3
Rural	543	44.7
Gender		
Male	607	50.0
Female	608	50.0
Educational level		
Low (primary education <10 years)	339	27.9
Medium (secondary education 10–12 years)	483	39.8
High^a^	393	32.3
Age groups (years)		
18–24	242	19.9
25–34	297	24.4
35–49	347	28.6
50–59	186	15.3
60–74	143	11.8

Legend: ^a^Tertiary education >12 years, completed by obtaining a diploma. University. Post-university.

### Design and sampling

#### Study setting

The study was conducted between 2011 and 2012 in urban areas within Bucharest, Craiova and Satu Mare, the villages of Cioroiaşi and Stolnici and in villages in the county of Satu Mare. When choosing these locations as statistical units, their socio-demographic and cultural characteristics were taken into account, including age of the settlement; population density; access by car, train and plane and rank according to the plan for national territory arrangement.

Subjects with a high level of education filled out the questionnaire themselves; interviewers later verified the responses in face-to-face encounters with the respondents. For subjects with low and medium levels of education, questionnaire-based face-to-face interviews were used. The response rate was 100%.

#### Measurements: questionnaire design

This study used a subset of items from an omnibus questionnaire of 96 items focusing on topics related to family functioning: economic function, cohesion and solidarity, education and sexual and reproductive life. Significance tests and anticipated model validation tests were based on Pearson chi-square statistics with 5–20 degrees of freedom.

In the present study I focused on nine items:

–What age were you when the following events took place: first menstruation/first spontaneous ejaculation… ; first intercourse …; if the event did not take place, please specify ‘not applicable’.

–Are you still in a couple with that person? (response values: ‘No’, ‘Yes’, ‘I am not yet sexually active’).

–When you first had intercourse, for how long had you known that person (with whom you had your first intercourse): …

–Was the first intercourse protected? (response categories: ‘No’, ‘Yes’; and with what did you protect yourself?…, ‘I am not yet sexually active’).

–How many sexual partners have you had so far? …

–What age were you when you first received information on matters of sex? …

–How much information have you received on matters of sex from the following sources: friends or acquaintances; parents or other relatives; doctors or health staff; specialized books; school; newspapers, radio, television or, the Internet; other sources, (please specify: …) (variants of answer: a lot, little, almost none).

–Did you benefit from sexual education classes in school? (response categories: ‘No’, ‘Yes’).

–To what extent have you discussed the following subjects with your parents: sexual abstinence before marriage; menstruation or first spontaneous ejaculation; how pregnancy occurs; prevention of pregnancy; abortion; sexually transmitted diseases; any sexual problem. The scale of talking with parents about sex and sexual health appeared to have good internal consistency (Cronbach’s alpha = 0.90). All items correlated to a good degree with the total scale (lower r = 0.52). After conducting a 50-subject pilot survey, we created the final version of the questionnaire. A three-point Likert scale was used in the questionnaire to accommodate the sample size. For the latent class analysis (LCA), the three-point scale was recoded into binary: ‘A lot’, coded 1, and ‘Little or almost nothing’, coded 0.

### Ethical considerations

Informed written consent was obtained from each participant at the time of recruitment. The subjects were informed that they could withdraw from the study at any stage, and they were assured of confidentiality. The study was approved by the Ethics Commission of the Francisc I. Rainer Anthropological Institute of the Romanian Academy.

I have used the services of eight qualified interviewers to collect and check the responses. All of the interviewers had expertise in the fields of sociology, psychology and medicine. To ensure that the respondents felt at ease and to guarantee accurate answers, the respondents were assigned interviewers of the same gender and as far as possible of similar age. Care and sensitivity were used at all times when dealing with the respondents. The interviews were held in specially designated rooms: offices of family physicians, rooms made available with the help of village mayors and university lecture rooms.

### Data management and statistical analysis

The Pearson chi-square tests and LCA statistical analysis were conducted using the statistical programs SPSS, Version 15 (SPSS Inc., Chicago, IL) and Latent Gold (Statistical Innovations Inc., Belmont, MA). LCA was employed to examine distinct patterns of received parental education about sex and sexual health among participants. Demographic variables used in the statistical analyses were environment, gender, level of education and age group.

## Results

### First intercourse

At the moment of investigation, 15 subjects (1.2%), of whom 13 were women, had not become sexually active. Among the 1200 subjects who were sexually active, age 18–19 was the most common time to have engaged in first intercourse (34.8%). At 30.1%, the percentage who were sexually active between 16 and 17 was only slightly lower, whereas 6.7% had FI between 14 and 15, and 0.5% had FI when they were younger than 14.

The average age at sexual initiation was 18.52 years in the total sample, with a range of 13– 35. The average age at FI was almost 1 year higher for women (18.97 years, compared with 18.08 years for men). The average age at FI was higher among urban respondents (18.72 years, compared with 18.27 years in rural areas) and for individuals with a higher level of education (18.94 years, compared with 18.07 years for those with low levels of education). The average age at FI decreased as respondent age increased. Focusing on the most extreme age groups (18–24 and 60–74), the average age at FI was 16.99 years (standard deviation = 1.40, range = 13–21) in the 1988–1994 birth cohort and 19.55 years (standard deviation = 3.49, range = 13–35) in the 1938–1952 birth cohort. In other words, the age at sexual initiation was over 2.5 years earlier for the youngest cohort compared with the oldest cohort.

In the age groups for which the statistical analyses in this study were conducted (ages 18–35 years and 36–74 years), the average of age at sexual initiation was 17.67 years (standard deviation = 2.18, range = 13–30) in the 1977–1994 birth cohort and 19.24 years (standard deviation = 2.8, range = 13–36) in the 1938–1976 birth cohort. This amounts to a decrease in the age at FI of over 1.5 years for the younger group of respondents.

No significant differences between urban and rural residents were found in the average age at FI, but the test statistic is close to significance at p = 0.051. Of subjects who lived in rural areas, 53.4% engaged in FI when they were 15 or younger and 36.4% were 25 or older at FI. Of urban residents, 46.6% engaged in FI at 15 or younger, and 63.6% were 25 or older at FI.

A higher proportion of men became sexually active before the age of 17 years than did women (chi-square = 48.29, p < 0.001) (Table 2). Among subjects engaging in FI at 15 or younger, the percentage with a low level of education was 14.8 percentage points higher than the percentage among subjects engaging in FI at 25 or older. Among those who were 15 or younger at FI, the percentage with a high level of education was 23.9 percentage points lower than the percentage of those aged 25 or older at FI with a high level of education (chi-square =29.38, p < 0.001) (Table 2).

**Table 2:** **Distribution of subjects by groups of age at FI, *a*ccording to socio-demographic characteristics**

**Age groups at FI**	Gender*		Educational level*			Age groups (years)*	
	**Male**	**Female**	**Low**	**Medium**	**High**	**18-35**	**36-74**
<=15	75.0	25.0	33.0	38.6	28.4	67.0	33.0
16–17	57.9	42.1	33.0	42.1	24.9	63.7	36.3
18–19	47.7	52.3	28.8	36.7	34.5	43.6	56.4
20–24	38.8	61.4	20.3	42.8	36.9	23.4	76.6
> = 25	40.9	59.1	18.2	29.5	52.3	22.7	77.3
Total	50.3	49.7	27.9	39.7	32.4	45.8	54.3

Legend: FI-first intercourse, *p < 0.001.

Compared with respondents engaging in FI at age 25 or older, a higher percentage of those engaging in FI at 15 or younger were 18–35 at the time of the study. In contrast, for those who engaged in FI at age 25 or older, the percentage aged 36–74 is higher than the percentage in that age group of those who were 15 or younger at FI (chi-square =131.26, p < 0.001) (Table 2).

### In a couple with the first sexual partner

Over 60% of the respondents in this study were not in a couple (officially married or in a consensual union) with the person with whom they were sexually active at the time of the data collection for this study.

The percentage of respondents who were in a couple with the same person with whom they had FI was higher for rural respondents than for urban respondents (45.7% of rural residents vs. 32.5% of urban residents; chi-square = 22.02, p < 0.001) and for women than for men (53.5% of women vs. 23.5% of men; chi-square = 114.22, p < 0.001).

The percentage of respondents who were in a couple with the same partner from their FI was higher among those aged 60–74 (48.3%) than among those aged 18–24 (35.5%; chi-square = 24.22, p < 0.001).

The ratio of those who married their first sexual partner was higher among subjects with low levels of education (44.8%) than among those with average and high levels of education (36.3%; chi-square =11.35, p < 0.01).

### Length of acquaintance with partner at first intercourse

Of the 1200 subjects who were sexually active, almost half engaged in FI between 1 month and 1 year after meeting their FI partner. Just under one-third (32.5%) of the subjects had known their FI partner for over a year, 12% had known the person for between 1 week and 1 month and 5.8% had known the person for less than a week.

The environment of residence had a statistically significant influence on the time acquainted with partner at FI (chi-square = 26.71, p < 0.001). The most conspicuous difference was found among those who had known their partner for less than a week, with the number of urban residents in this group being over three times higher than the number of rural residents. However, urban respondents were also well represented among those reporting having FI with a person they had known for a longer time (over a month) (Table 3). Those who knew their partners longer at FI were more often women (chi-square = 63.65, p < 0.001). Notably, over 6.5 times more men than women engaged in FI less than a week after meeting their partner (Table 3).

**Table 3:** Distribution of subjects regarding being acquainted with their partner at FI, depending on socio-demographic characteristics

**For how long had they known the person with whom they had FI**	Environment*		Gender*		Educational level*		
	**Urban**	**Rural**	**Male**	**Female**	**Low**	**Medium**	**High**
Less than a week	77.1	22.9	87.1	12.9	22.9	44.3	32.9
A week – a month	40.4	59.6	64.4	35.6	47.3	28.8	24.0
A month – a year	55.6	44.4	48.7	51.3	26.3	40.4	33.3
Over a year	56.2	43.8	41.0	59.0	24.1	41.8	34.1
Total	55.2	44.8	50.3	49.7	27.9	39.7	32.4

Legend: FI-first intercourse,* p < 0.001.

Educational level also had a statistically significant influence on the length of time acquainted with partner at FI (chi-square = 31.81, p < 0.001). Those with average or higher levels of education made up the highest proportions of the group who had FI with a person they had known for less than a week. However, among those who had known their first sexual partner between a month and a year or over a year, the proportions were highest for those with average or higher levels of education (Table 3).

No statistically significant differences were found between the different age groups of subjects in terms of the length of acquaintance with partner at FI.

### Protection at first intercourse

The use of protection at FI was very low; only 23.7% of the 1200 subjects who were sexually active used protection. Condoms were the main method of protection at FI (20.6%, N = 250). Both men and women, in similar proportions, failed to use protection against STIs or pregnancy at first intercourse. Of subjects who used protection at FI, the proportion who were urban residents was significantly higher than the proportion who were rural residents (chi-square = 35.01, p < 0.001). The proportions of this group with average and high levels of education were also higher than the proportions with low levels of education (chi-square = 25.72, p < 0.001). The largest statistically significant differences regarding use of protection at FI were found between age groups; among those who had protected themselves at FI, the percentage of subjects who were aged 18–35 at the time of the study was almost three times higher compared with the percentage aged 36–74 (chi-square = 122.15, p < 0.001) (Table 4).

**Table 4:** Distribution of subjects regarding protection at FI, depending on socio-demographic characteristics

**Protection at FI**	Environment *		Educational level*			Age groups (years)*	
	**Urban**	**Rural**	**Low**	**Medium**	**High**	**18-35**	**36-74**
No	50.4	49.6	31.4	38.6	29.9	36.9	63.1
Yes	70.4	29.6	16.5	43.0	40.5	74.3	25.7
Total	55.2	44.8	27.9	39.7	32.4	45.8	54.3

Legend: FI-first intercourse, * p < 0.001.

### Number of sexual partners

The average number of sexual partners was 4.48 overall (median = 3, maximum = 80), 6.56 for men (median = 4, maximum = 80) and 2.37 for women (median = 1, maximum = 19). It is noteworthy that 22 men insisted on answering this question using expressions such as: ‘numberless’, with the mathematical sign for infinity or by saying ‘so many that I cannot remember’ or ‘The hell knows; I did not pass up any opportunities’.

The average number of sexual partners was higher in the urban environment (5.07, compared with 3.75 in the rural environment). The average number of sexual partners was similar across age groups (4.37 for subjects aged 18–35 and 4.57 for subjects aged 36–74). Focusing on the most extreme age groups (18–24 and 60–74), the average number of sexual partners was 3.57 (median = 2, maximum = 25) for the 1988–1994 birth cohort and 4.44 (median = 2, maximum = 30) for the 1938–1952 birth cohort. The average number of sexual partners was higher among subjects with high levels of education (mean = 4.56, median = 3, maximum = 30) than among those with low levels of education (mean = 3.94, median = 2, maximum = 80).

Among those who had more than three sexual partners, the percentage of urban residents was higher than the percentage of rural residents (chi-square = 50.44, p < 0.001), the percentage of men was higher than the percentage of women (chi-square = 279.44, p < 0.001), the percentages with average and high levels of education were higher than the percentage with less education (chi-square =18.76, p < 0.05) and the percentage aged 36–74 was higher than the percentage aged 18–35 (chi-square = 20.50, p ≤ 0.01) (Table 5).

**Table 5:** Distribution of subjects regarding the number of sexual partners, depending on socio-demographic characteristics

**Number of sexual partners**	Environment*		Gender*		Educational level**			Age groups (years)***	
	**Urban**	**Rural**	**Male**	**Female**	**Low**	**Medium**	**High**	**18-35**	**36-74**
1	44.0	56.0	21.9	78.1	32.8	35.7	31.5	41.4	58.6
2	46.5	53.5	39.0	61.0	33.7	37.4	28.9	57.8	42.2
3	63.9	36.1	54.4	45.6	25.6	38.9	35.6	45.6	54.4
4-10	63.5	36.5	74.4	25.6	22.8	43.2	34.0	46.2	53.8
11-20	75.0	25.0	86.7	13.3	23.3	46.7	30.0	30.0	70.0
>20	60.0	40.0	100	0	13.3	53.3	33.3	53.3	46.7
Total	55.2	44.8	50.3	49.7	27.9	39.7	32.4	45.8	54.3

*p < 0.001; **p < 0.05; ***p < 0.01.

### First information on sex

Of the entire sample (N = 1215), 44.2% first received information on sex when they were older than 15 (mean = 15.39, median = 15, range = 6–25, standard deviation = 2.53). The age of 14 had the highest frequency (21.8%). The age at which subjects first received information about sex was correlated with the age at first spontaneous ejaculation (N = 549, mean = 14.25, median = 14, standard deviation = 1.42, range = 9–18) or menarche (N = 609, mean = 13.56, median = 14, standard deviation = 1.38, range = 9–19) (chi-square = 157.62, p < 0.001). A larger percentage of urban residents, compared with rural residents, first received information about sex before they were 15 (chi-square = 68.54, p < 0.001).

Although significant differences were found by gender regarding the age at which the subjects first received information about sex (chi-square = 15.94, p < 0.001), no clear pattern was identified. Two differences by gender are particularly conspicuous. Many more men than women first received information on sex at 13 or 15, and most respondents who first received information on sex at age 14 were women. This is an aspect which requires further investigation.

More respondents with a low level of education reported that they first received information on sex later than those with average and higher levels of education (chi-square = 71.06, p < 0.001) (Table 6). Younger subjects made up a larger percentage of those who first received information on sex earlier than did older subjects (chi-square = 81.84, p < 0.001) (Table 6).

**Table 6:** The age of subjects at which they received the first information on sex, by socio-demographic characteristics

**Age groups of first information on sex**	Environment*		Gender*		Educational level*			Age groups (years)*	
	**Urban**	**Rural**	**Male**	**Female**	**Low**	**Medium**	**High**	**18-35**	**36-74**
<13	76.6	23.4	47.7	52.3	13.5	36.0	50.5	65.8	34.2
13	70.8	29.2	60.0	40.0	10.8	55.8	33.3	62.5	37.5
14	64.9	35.1	43.4	56.6	21.9	42.3	35.8	59.2	40.8
15	48.9	51.1	58.8	41.2	28.0	39.0	33.0	42.3	57.7
>15	44.9	55.1	48.2	51.8	37.6	35.9	26.4	33.9	66.1
Total	55.3	44.7	49.9	50.1	27.9	39.8	32.3	46.4	53.6

*p < 0.001.

### Sources of information on sex

The smallest part of information on sexual behaviour (no more than 10%) came from the school, doctors or health staff.

Friends and acquaintances were the most frequently reported source of information on sexual behaviour (35.2%). The mass media and the Internet were the next most frequently reported sources (26.4%), followed by specialized books (22.7%). Only 11 subjects stated that they learnt about sexual behaviour from partners, personal practice and experience.

From all of the sources of information on sex offered as response values, 41.1% (N = 499) of the subjects received information ‘little or not at all’. Only 2.1% of the subjects received ‘a lot’ of information from all six sources of information offered as response values.

Among those who had received ‘a lot’ of information on sex from sources such as doctors and health staff (8.4%, N = 102), the highest percentages were recorded for the individuals from urban environments (chi-square = 13.39, p < 0.001), for women (chi-square = 9.47, p < 0.01), for people with average and high levels of education (chi-square = 19.47, p < 0.001) and for subjects aged 18–35 (chi-square = 24.07, p < 0.001) (Table 7).

**Table 7:** Distribution of subjects regarding the sources of information on sex, depending on socio-demographic characteristics

**Sources of information on sex**		**Environment**		**Gender**		**Educational level**			**Age groups (years)**	
		**Urban**	**Rural**	**Male**	**Female**	**Low**	**Medium**	**High**	**18-35**	**36-74**
Parents, relatives	A little^a^	53.4*	46.6*	52.1*	47.9*	29.1**	38.6**	32.3**	43.6**	56.4*
	A lot	69.5*	30.5*	32.6*	67.4*	19.1**	48.2**	32.6**	68.1*	31.9*
Doctors, sanitary staff	A little^a^	53.7*	46.3*	51.2**	48.8**	29.6*	39.3*	31.2*	44.3*	55.7*
	A lot	72.5*	27.5*	35.3**	64.7**	9.8*	45.1*	45.1*	69.6*	30.4*
Specialized books	A little^a^	49.1	50.9	53.6	46.4	34.7	39.0	26.3	45.5***	54.5***
	A lot	76.4*	23.6*	37.3*	62.7*	4.7*	42.4*	52.9*	49.6***	50.4***
Newspaper, radio, TV, internet	A little^a^	47.8*	52.2*	50.4***	49.6***	35.0*	38.3*	26.7*	37.2*	62.8*
	A lot	76.3*	23.7*	48.3***	51.7***	8.1*	43.9*	48.0*	72.0*	28.0*
Friends, acquaint-ances	A little^a^	53.5***	46.5***	44.9	55.1	27.1***	39.3***	33.7***	38.9*	61.1*
	A lot	58.6***	41.4***	59.1*	40.9*	29.4***	40.7***	29.9***	60.3*	39.7*
School	A little^a^	55.4***	44.6***	50.5***	49.5***	29.8*	37.5*	32.7*	43.7*	56.3*
	A lot	54.5***	45.5***	43.8***	56.2***	10.7*	60.3*	28.9*	71.1*	28.9*

^a^Little, almost none; *p < 0.001; **p < 0.005; ***p > 0.5.

Among the subjects who had learned ‘a lot’ of information on sex from sources such as newspapers, radio, TV or Internet (26.4%, N = 321), the highest percentages were recorded for those from urban environments (chi-square = 77.95, p < 0.001), for those with average and high levels of education (chi-square =96.165, p < 0.001) and for subjects aged 18–35 (chi-square = 114.44, p < 0.001; Table 7).

Among those who gathered ‘a lot’ of information on sex from friends and acquaintances (35.2%, N = 428), the highest percentages were recorded for men (chi-square = 22.544, p < 0.001) and for subjects aged 18–35 (chi-square = 51.04, p < 0.001; Table 7).

### Sexual education in school

Under one-third (25.9%, N = 315) of subjects benefited from sexual education classes in school. The percentage benefiting from sexual education classes in school was higher among urban subjects (compared with rural subjects, chi-square = 15.37, p < 0.001), women (compared with men, chi-square = 8.38, p = 0.004), those with average and high levels of education (compared with those with less education, chi-square = 45.77, p < 0.001) and those aged 18–35 (compared with those aged 36–74, chi-square = 118.07, p < 0.001) (Table 8).

**Table 8:** Distribution of subjects regarding sexual education in school, depending on socio-demographic characteristics

**Did you benefit from sexual education classes in school?**	Environment*		Gender*		Educational level*			Age groups (years)*	
	**Urban**	**Rural**	**Male**	**Female**	**Low**	**Medium**	**High**	**18-35**	**36-74**
No	52.0	48.0	52.3	47.7	32.6	35.1	32.3	37.2	62.8
Yes	64.8	35.2	42.9	57.1	14.6	53.0	32.4	72.7	27.3
Total	55.3	44.7	49.9	50.1	27.9	39.8	32.3	46.4	53.6

*p < 0.001.

### Communication with parents regarding SRH

Only 4.2% (N = 51) of subjects had discussed ‘a lot’ with their parents about all seven variants of answers: sexual abstinence before marriage, any sexual problem, sexually transmitted diseases, menstruation/first spontaneous ejaculation, how pregnancy occurs, prevention of pregnancy and abortion. The majority of subjects (70.5%, N = 857) communicated with their parents ‘little or almost not at all’ on SRH-related subjects.

For those who discussed sex with their parents ‘a lot’, the most prevalent subject was menstruation/first spontaneous ejaculation (18.4%), followed by the prevention of pregnancy (15.9%) and how pregnancy occurs (15.3%).

In terms of sexual education received from parents, the LCA showed that the most suitable model is a division into five clusters representing types of education. Seven items (binary indicator variables) were used, with responses indicating that topics were discussed with the parents ‘a lot’ (item response coded 1) or ‘little or almost not at all’ (item response coded 0). For the analysis, the 1200 subjects who had already engaged in FI were selected. The optimal model was determined using the minimum value of the Bayesian Information Criteria statistics (BIC = 4178). The small values of the bivariate residuals provided a direct check of the local independencies assumption. Comparisons were made between the clusters using the conditional probabilities presented in Table 9. A conditional probability (p) close or equal to 1.0 indicates that members in the relevant class endorse a category item response.

**Table 9:** Profiles of communication with parents on SRH (LCA - conditional probabilities)

	**Poor absent**	**Limited minimal**	**Complex extensive**	**Pregnancy oriented**	**STD oriented**
	**Cluster1**	**Cluster2**	**Cluster3**	**Cluster4**	**Cluster5**
Cluster size	0.743	0.099	0.060	0.053	0.044
Indicators					
Sexual abstinence before marriage					
Little or almost none	0.99	0.56	0.26	0.71	0.81
A lot	0.01	0.44	0.74	0.29	0.19
Menstruation/pollution					
Little or almost none	0.98	0.47	0.02	0.09	0.85
A lot	0.02	0.53	0.98	0.91	0.15
How pregnancies appear					
Little or almost none	0.99	0.78	0.03	0.08	0.64
A lot	0.01	0.22	0.97	0.92	0.36
Prevention of pregnancies					
Little or almost none	0.99	0.91	0.03	0.05	0.44
A lot	0.01	0.09	0.97	0.95	0.56
Abortion					
Little or almost none	1.00	0.97	0.00	0.31	0.76
A lot	0.00	0.03	1.00	0.69	0.24
STIs					
Little or almost none	0.98	0.95	0.00	0.84	0.05
A lot	0.02	0.05	1.00	0.16	0.95
Any sexual problem					
Little or almost none	1.00	1.00	0.01	0.91	0.60
A lot	0.00	0.00	0.99	0.09	0.40

Cluster 1 (**Poor or absent**) is the most numerous, comprising 74.3% of the subjects, where the sexual education received from parents is little or absent. Almost all the topics relating to sex and sexual health were discussed with the subjects’ parents, but to a small extent or almost not at all. Of subjects in this cluster, only 20.3% had used protection at FI.

In Cluster 2 (**Limited or minimal**), the education received is minimal and limited. In this cluster, issues related to sexual abstinence (p = 0.44) and puberty (p = 0.53) were covered, but with minimal probabilities. This cluster includes 9.9% of the subjects. In this cluster, only 23.5% used protection at FI.

In Cluster 3 (**Complex or extensive**), the probability of approaching all of the sexual topics is high (almost equal to 1), although there is perhaps less coverage of the issue of sexual abstinence, where a p = 0.74 was reported. This cluster includes 6.0% of the subjects. Women make up 77.8% of this cluster, and the cluster is more present in the urban environment (66.7%) than in the rural environment. In this cluster, 38.9% used protection at FI.

In Cluster 4 (**Pregnancy oriented**), there was a higher probability of discussing related subjects: menstruation/first spontaneous ejaculation (p = 0.91), pregnancy (p = 0.92) and avoidance of pregnancy (p = 0.95). To a certain extent, the question of abortion was also discussed in this cluster (p = n0.69). The cluster includes 5.3% of the subjects. The proportion of women in this cluster is 85.9%, and the cluster is more present in rural environment (62.5%) than in the urban environment. In this cluster, 39.1% used protection at FI.

Cluster 5 (**STIs Oriented**) is the segment in which only the approach of STI-related problems has a strong probability (p = 0.95). The cluster includes 4.4% of the subjects. The proportion of men in this cluster is 60.4%, and the cluster is more present in the urban environment (69.8%) than in the rural environment. This cluster has the highest proportion of individuals who used protection at FI (41.5%).

## Discussion

The results of the study show that Romania, similar to many other post-communist Eastern European countries, has caught up with Western countries in terms of issues of sexual behaviour, as evidenced by a decrease of the age at sexual initiation, more intense premarital sexual activity, a general liberalisation of values and attitudes towards sex and an actual ‘sex revolution’. The main problems observed in Romania are a weaker implementation rate of SRH education and insufficiently dealing with the psychological and emotional elements of a relationship in education and teaching.

### First intercourse

Many young girls do not know how to negotiate the start of sexual life and agree to have sex, sometimes without expressly wanting to do so, for fear of being abandoned. Many teenagers who became sexually active early, without being ready for it, have expressed regret and a lack of enjoyment of their unique experience. Most of them acted under the psychological pressure of peer group norms to be accepted and valued [29].

Compared with the findings from an American study that used data collected from teenagers in 2005 and 2007 the prevalence of early sex, defined as having first sexual intercourse before age 14, is much lower in Romania; the prevalence of early sex was more than one-third in the American study, compared with 0.5 in the present Romanian study [30].

As for the prevalence of early sex in men, defined as having first sexual intercourse before age 15, with the present study shows a similar prevalence as that reported in an African study from 2003. In rural areas, the percentage of subjects who engaged in FI before the age of 15 was 13.1% among men aged 15–24 in the African study and 12.7% among men aged 18–24 in the present Romanian study [31]. Further, the findings from the present study on the average age at FI among young people aged 15–24 are almost equal to the findings of a 2012 Ethiopian study (17.07 years in the Ethiopian study vs. 16.99 years in the present Romanian study) [32].

Similar to the findings of other studies, this study shows that women waited longer before having their first sexual experience [33]. This could be explained by several aspects. First, young men are encouraged to lose their virginity and to have as many sexual partners as possible as a sign of virility and manhood. On the contrary, girls are encouraged to postpone as much as possible the moment of their first sexual act and to have a small number of partners as a proof of sobriety and obedience. At the same time, girls tend to prioritize the emotional relationship higher than the sexual relationship [34].

Similar to other studies, we identified a difference in patterns of sexual activity by birth cohort [35-37]. The average age for becoming sexually active was almost 2.5 years younger for the 1988–1994 birth cohort younger (average age of FI almost 17 years) than for those born between 1938 and 1976.

### In a couple with the first sexual partner

Studies carried out after 1990 in Europe, the United States and other parts of North America have shown that the proportion of those who considered that premarital sex was wrong has decreased by almost 90–50% [38].

Less than half of the respondents within this study were in a couple (officially married or in a consensual union) with the same partner with whom they had started their sexual life. The percentage of those who were in a couple with their first sexual partner was 13.2 percentage points higher among rural residents than urban residents, more than double in women compared with men, 12.8 percentage points higher among the 1938–1952 birth cohort than in the 1988–1994 birth cohort and 8.5% higher among those with a low level of education compared with those with more education. In our sample, there were men who were in a couple with their first sexual partner, but public opinion usually does not associate FI with marriage in men. The high number of women reporting being in a couple with their FI partner may demonstrate a ‘cover-up’ story.

In the communist period, although people explicitly talked about gender equality, it was considered that men, because of their biological characteristics, could not resist the temptations offered by women, so sexual abstinence before marriage and marital fidelity were women’s responsibility. The finding that men have a lower age of sexual initiation and a higher number of sexual partners, may be interpreted as the result of under-reporting by women and over-reporting by men. This is an effect of societal judgment of sexual behaviour by a gendered double standard [39].

### Length of acquaintance with partner at first intercourse

Not knowing one’s sex partner well enough increases the risk of exposure to an STI. Studies demonstrate that STIs are associated with not being familiar with partner’s behaviour [40,41]. In the analysed sample, individuals who had known their partner at FI for a between a month and over a year predominated. This study has clearly shown that that the proportion of men who knew the person with whom they had FI for less than a week at the time of FI was over two times higher for urban subjects than for rural subjects.

For good and safe sex, it is necessary to know yourself and your partner. However invasive the question of ‘how many sexual partners have you had so far’ might seem, it was necessary to ask it, not to uncover the subjects’ values and morals, but to protect them against STIs. Having even one partner is sufficient to contract a STI, if that partner is infected. Therefore, knowledge on this aspect of behaviour is required to educate sexually active persons about the dangers of entering a sexual relationship without really knowing one’s partner. Stressing the importance of medical analyses for checking a partner’s STI status when entering a relationship (and with the essentiality of using a condom until the analysis results are obtained) should be aimed at achieving a preventive behaviour without conveying offensiveness or a lack of trust.

### Protection at first sexual intercourse

In Romania, over 22 years have passed since the introduction and approval of large scale use of contraceptives and protection against STIs. People who were 35 years old in 2012 were born in 1977, so, even if they became sexually active at age 13, they have had every possibility to practice protected sex. Given this, the percentage of subjects aged 18–35 who did not use any protection at FI despite the fact that they could have done so (61.6%) are alarmingly high.

The percentage of people who had protected sex at FI is more than two times larger in the urban environment compared with the rural environment and among those with average or high levels of education compared with those with less education. The percentage who had protected sex at FI is almost three times higher among the subjects aged 18–35 than among those aged 36–74.

When compared with previous studies conducted in Romania (RRHS 1999, RHSR 2004 and the 2005–2006 Rada–Tarcea Study) [18,19,21], the present study shows a slight increase in the prevalence condom use among the population aged 18–35. Similar to findings from other studies, rural residents in this study environment reported less use of protection against STIs, HIV or unwanted pregnancy [42]. Previous studies have identified that potential condom users raised the question of whether the condom could reduce the pleasure of sexual intercourse [43,44]. Therefore, one of the methods of encouraging protection could be the stronger highlighting of advantages and positive consequences in the long run of using the condom with occasional partners, including protecting against the risks of unsafe sex. At the same time, it is necessary to increase men’s responsibility regarding SRH and to educate women to negotiate the use of condoms.

A British study conducted in 1990 by interviewing 96 respondents ascertained that half of the subjects knew the partner at FI for two weeks or less and one-fourth of the subjects knew the partner at FI for only 24 hours. The percentages of subjects who knew or did not know anything about the FI partner were relatively equal.

Condom use was more frequent among older women, who after the sexual act progressed later on in their relationships [45]. Comparing the results of the present study with the results of the British study, the subjects from Romania engaged in FI after knowing the partner for a longer period of time, but condom use was low among the subjects in both studies.

### Number of sexual partners

An American study conducted in 2007 on 100 subjects aged 18–40 identified the mean number of vaginal intercourse sexual partners as eight (median = 4), with men reporting a mean of 13 (median = 6) and women reporting a mean of five (median = 3) [46]. The average number of sexual partners found in the present study was just over half that found in the American study.

Among those who had only one partner, most of them ranged between 36 and 74 years old, although it was theoretically possible for them to acquire a higher number of sexual partners. First, we must take into account the lower age average at marriage in this sample compared with national and international statistics [8]. Second, we must consider the frankness of answers, considering the traditional society which is less permissive to premarital sex or sex outside marriage. In light of the fact that women in this study reported having only one sexual partner 3.5 times more frequently and having two sexual partners over 1.5 times more frequently than men, it is also necessary to consider gender-related aspects. Similar to the lower average age of the sexual initiation and the higher average number of sexual partners for men, the results have been interpreted from the biological and cultural point of view [47]. One explanation is that, for men, sexual success is the means by which they obtain a prestigious position among other men [48]. Lise Eliot argues that biological gender differences are minor and that socialization is a much greater force in the creation of a gendered world; it is true that men and women are different, but the environment offers a shape and stresses these differences [49].

### First information on sex

There is no standard suitable age at which to initiate discussions about sex with a child. Learning in this sphere is gradual. The fact that more than half of the subjects of this study first received information on sex before they were 15 seems to be a positive fact, provided that this information comes from relevant sources, an aspect which will be explored below.

Further studies are needed based on the influence of educational level and gender on first information about sex, because no clear patterns were identified in this study. The fact that younger subjects first received information about sex earlier than did older subjects indicates a change in the attitude and behaviour patterns related to sex, a taboo subject in traditional society and an embarrassing topic in the communist period.

### Sources of information on sex

In line with previous research [50], this study identified friends and acquaintances as the most common sources of information on the subject of sexual behaviour. Over one-fourth of the sample reported mass media as a source of information, and pertinent sources such as school, doctors, and health staff, are very poorly represented. Compared with the 1999 RRHS, we identified an increase of the contribution of doctors and mass media and a decrease of the contribution of friends and acquaintances.

Age group has a statistically significant impact on all of the sources of information on sex except for specialized books. Younger people (those aged 18–35) more commonly reported the remaining five sources of information, compared with older people aged 36–74.

Before 1989, young people in Romania, unlike most of their Western counterparts, did not receive sufficient sexual education, and there was no contraceptive education. The aggressively pro-natalist policy during the Ceausescu era prohibited the sale and use of modern contraceptives and abortion. The proportions citing the more pertinent sources of information such as doctors, health staff and specialized books were, on average, almost three times higher among urban residents than among rural residents, almost two times higher among women than men and over six times higher among subjects with average or high levels of education than among those with lower levels of education. Male subjects more often tended to be informed about sex from less pertinent sources. The proportions drawing on parents and relatives as sources of information on sex were over two times higher in urban environments and among female subjects, signalling greater opportunities for these groups in the family environment. However, no matter how well intended they may be, parents and relatives do not always provide accurate information. Nevertheless, previous studies have shown that, when parents discuss SRH with their children and monitor their children’s behaviour, the children are less likely to exhibit risky behaviour, both in general and in sexual matters in particular [51].

E-health (health care, offering health-related information on the Internet) must be regarded as an opportunity to improve health care services. Surfing the Internet to learn more about sexual health is increasingly preferred, because it has a series of advantages such as: no or very low costs, non-discrimination, maintaining anonymity, no requirement of travel and ease of accessibility in terms of time and place. Similar to previous work [52], this study has shown, that rural residents, those with a low level of education and older persons turn less frequently to the Internet as a source of information. The greatest challenge for e-health is the accuracy and reliability of information. The Internet is teeming with inaccurate, incomplete information that can lead to making wrong decisions [53].

For instance, there are many Romanian websites dedicated to the positive effects of sexual activity on health. This is a positive change, considering that the topic of sexual activity, especially sexual activity without a reproductive purpose, used to be taboo. These sites are Romanian translations of scholarly or popular science articles, but the presentations on such websites tend to be very imbalanced because they conclude their account with a single, possibly optional, sentence about safety; for instance, ‘one should not hesitate when the opportunity arises, and the magic (author’s note: sexual act) can happen in full safety’. Nothing is said about the importance of aspects such as the emotional bonding of a couple, commitment in a relationship or multiple partners.

Therefore, a goal to be pursued might be a more serious approach to dealing with the observance of laws and the higher ethics of presentation and organization of information, including careful checking of online material by qualified teachers and counsellors. A good example in this respect is ROmedic, a Romanian portal for online medical information, set up for doctors and for whomever may be interested in health, including precise rules of use and ethics [54].

### Sexual education in school

Over three-quarters of the respondents to the 1999 RRHS indicated that the optimal age for receiving school classes about how pregnancy occurs and about contraception and STIs was younger than 15 years [18]. Therefore, the awareness of the importance of sexual education in schools is present. The fact that just under 30% of the subjects of this study benefited in school from sexual education classes indicates the continuing need to meet this demand. Sexual education in school was reported to have been available almost two times more frequently for those in urban areas, about 14% more often for women, about three times more for individuals with average or high levels of education and over 2.5 times more by younger people.

The government with its political regime and the church have exerted a large influence on sexual education. Integrated in other teaching subjects or in separate, independent courses under various names, sexual education has been mandatory in schools since 1970 in countries such as Austria, Denmark and Finland; since 1990 in Germany; since 1995 in Greece; since 1996 in Slovakia and since 2001 in France. There are no official laws regarding sexual education in Italy, and sexual education is not mandatory in the United Kingdom, Bulgaria, Poland or Cyprus [55].

In Romania, after 1990, volunteers were trained by international agencies and non-governmental organizations to teach lessons in high schools on the health of reproduction, family planning and STIs. Hence, sexual education was not systematically introduced in the curriculum. Starting with the school year 2004–2005, the optional subject *Education for Health* was introduced in the curriculum for classes I–XII. The teaching line is organized in a six-module structure (one module is studied over 2 school years), and each module has a chapter dedicated to *the health of reproduction and family.* In each module, the emphasis is placed on various topics, depending on the age of the students [56]. In the 2011–2012 school year, only about 12% of the students attended classes for this subject matter. The following statements of respondents, high school graduates aged 19, serve to highlight the dissimilarity between different school contexts:

‘As we were a high school specializing in economics, we had no classes for education for health at all, and even less sexual education’.

‘We learnt something about the human body in biology classes’.

‘We learnt ‘Education about Health’ in the tutoring class and a few times a year some NGOs came and held courses for the promotion of health’.

Considering that this teaching programme had not achieved its goal, in 2013 it was decided to make it a mandatory subject matter in school. However, it is still an action under the debate in the two chambers of Parliament of Romania.

### Communication with parents about SRH

In this study, communication with parents on questions relating to SRH was very scarce; less than 30% of the subjects discussed with their parents ‘a lot’ on the matters of SRH, the favourite topics being the menstruation/first spontaneous ejaculation and the occurrence and prevention of pregnancy. The percentages found in the present study regarding discussion with parents on SRH are much lower compared with other studies [57].

The discussions with parents about any sexual problem, STIs, menstruation/first spontaneous ejaculation, how pregnancy occurs and prevention of pregnancy and abortion, happened ‘a lot’ mainly in case of urban subjects, women, people with average or high levels of education and subjects aged 18–35. The trends are similar to those recorded in the 1999 RRHS.

Conversations with teenagers about sex depend chiefly on the established parent-teenager relationship and on the approach of the family to sex and sexuality. From the psychological point of view, the discussions aimed at information, guidance and support in matters of sex must be initiated first of all by the parents and next by the school. Unfortunately, parents still feel uncomfortable talking to their children about sex, sexuality and gender.

## Conclusions

Taking into account the fact that over 80% of the subjects engaged in FI at an interval between a month and over a year after meeting their partners, and taking into consideration the quite small average number of sexual partners, we can say that occasional sex is not a characteristic in the analysed sample, which is a positive aspect.

Taking into account risk behaviours for SRH such as FI with no protection, duration of partnership before FI of less than a month, and age of FI 17 years or less, it has been found that, on average, at least one of these risk behaviours was found among respondents in the rural environment, in men and in those with low and average educational level.

The difficulty of obtaining information about SRH for people younger than 14, the absence of sexual education classes in schools, poor information from specialists and schools and poor communication with parents regarding SRH are weak points identified in the entire sample, with particularly poor results for rural residents, men and those with a low or average level of education.

Compared with previous studies conducted in Romania, the present study shows only a slight improvement in obtaining information on sex from specialists in the field, from school and parents. Diminishing rates of teenage and unwanted pregnancy as well as the STIs rate, requires a multidisciplinary approach to prevention programmes. Both schools and parents are crucial factors in this extensive undertaking on SRH education programmes.

Additionally, there are specific reasons to improve SRH education in Romania. In the communist bloc, Romania developed and implemented the most restrictive legislation regarding access to the voluntary interruption of pregnancy, together with the interdiction of the import and sale of modern contraceptives. Women had to pass gynaecological examination at their workplaces every 1–3 months. The aim of this examination was to discover any possible pregnancy, so as to bar any potential attempts to illegally abort unwanted pregnancies. Such examinations were conducted in inappropriate medical conditions, and the women involved have described feeling like a herd driven to the slaughterhouse. This is one of the reasons that many women in Romania have shunned gynaecological examinations, a behaviour pattern they seem to have transmitted to their own offspring. Moreover, the anti-HPV vaccination campaign initiated in Romania in 2008, with fourth-grade school girls (average age of 11 years), failed because many parents declined vaccination of their daughters [23].

### Limitations

This study used retrospective data collection, and the distance in time since the premarital sexual experience and since using sources of information on sex can alter the way the subjects recalled these events.

This study did not include a representative sample, so we are not in a position to generalise our results.

This study did not analyse the impact of other factors such as biological factors, religion, health care professionals, the law, the availability of reproductive and sexual health services, the consumption of alcohol, the consumption of drugs or personality traits on sexual behaviour.

Pilot surveys and previous research have shown that subjects with low and medium levels of education, especially in rural areas, generally do not have the ability to complete questionnaires, so having all respondents fill out the questionnaires by themselves was not an option. Most subjects with higher levels of education have commented: ‘We do not need anybody to do the interview with us; we know better how to fill [out the questionnaire]’. Therefore, we had to give up questionnaire-based face-to-face interviews with such subjects. These two different modes of data collection (questionnaire-based face-to-face interviews for subjects with low and medium levels of education and self-completion for subjects with a higher levels of education) are likely to have introduced bias. However, because interviewers later checked the responses in a face-to-face meeting with the respondents, the bias may be insignificant.

## Abbreviations

HIV: Human immunodeficiency virus; STIs: Sexually transmitted infections; FI: First intercourse; SRH: Sexual and reproductive health; RRHS 1999: Romania Reproductive Health Survey 1999; RHSR 2004: Reproductive Health Survey Romania 2004; LCA: Latent class analysis.

## Competing interests

The author declares that she has no competing interests.

## Authors’ information

CR (BA in psycho-sociology and PhD in medicine) is a scientific researcher at the *Francisc I. Rainer* Institute of Anthropology, Romanian Academy, Biomedical Department, and an Associate Professor at the Spiru Haret University, Faculty of Sociology-Psychology; she teaches Psychology of Families and Couples and Social Psychology. She is also a clinical psychologist and psychotherapist and works in private practice. CR has accumulated experience from extensive research in the areas of sexual and reproductive health and family health.
